# Different domains of self-reported physical activity and risk of type 2 diabetes in a population-based Swedish cohort: the Malmö diet and Cancer study

**DOI:** 10.1186/s12889-020-8344-2

**Published:** 2020-02-21

**Authors:** Pascal M. Mutie, Isabel Drake, Ulrika Ericson, Stanley Teleka, Christina-Alexandra Schulz, Tanja Stocks, Emily Sonestedt

**Affiliations:** 1 Department of Clinical Sciences Malmö, Lund University, Malmö, Sweden; 2 Department of Clinical Sciences Lund, Lund University, Lund, Sweden; 3 Department of Nutrition and Food Sciences, Nutritional Epidemiology, University of Bonn, Bonn, Germany

**Keywords:** Diabetes, Physical activity, Domains

## Abstract

**Background:**

While a dose-response relationship between physical activity and risk of diabetes has been demonstrated, few studies have assessed the relative importance of different measures of physical activity on diabetes risk. The aim was to examine the association between different self-reported measures of physical activity and risk of type 2 diabetes in a prospective cohort study.

**Methods:**

Out of 26,615 adults (45–74 years, 60% women) in the population-based Swedish Malmö Diet and Cancer Study cohort, 3791 type 2 diabetes cases were identified from registers during 17 years of follow-up. Leisure-time (17 activities), occupational and domestic physical activity were assessed through a questionnaire, and these and total physical activity were investigated in relation to type 2 diabetes risk.

**Results:**

All physical activity measures showed weak to modest associations with type 2 diabetes risk. The strongest association was found in the lower end of leisure-time physical activity in dose-response analysis at levels approximately below 22 MET-hrs/week (300 min/week) representing around 40% of the population. Compared with the lowest quintile, the moderate leisure-time physical activity category had a 28% (95% CI: 0.71, 0.87) decreased risk of type 2 diabetes. Total physical activity showed a similar, but weaker, association with diabetes risk as to that of leisure-time physical activity. Domestic physical activity was positively and linearly related to diabetes risk, HR = 1.11 (95% CI: 0.99, 1.25) comparing highest to lowest quintile. There was no association between occupational physical activity and diabetes risk.

**Conclusion:**

A curvilinear association was observed between leisure-time physical activity and risk of diabetes. Beyond a threshold level of approximately 22 MET-hrs/week or 300 min/week, no additional risk reduction was observed with increase in physical activity.

## Background

Type 2 diabetes (T2D) is a chronic and complex metabolic disease characterized mainly by a relative insulin resistance or inadequate secretion. Uncontrolled, it is associated with high morbidity and mortality worldwide. In 2015, there were 415 million cases of T2D and this number is projected to increase to 642 million by 2040 if the same trend of incidence continues [1]. Excess adiposity, physical inactivity, genetics, poor nutrition, smoking and low socioeconomic status are main risk factors for developing T2D, the most common form of diabetes [1–4].

Physical activity has been associated with reduced risk of T2D in different populations [5–16], and the World Health Organization (WHO) recommends at least 150 min per week of moderate physical activity for health benefits [17]. Although many studies have demonstrated this inverse relationship between physical activity and risk of T2D, they have applied different metrics in quantifying physical activity and also used different domains (e.g., occupational, leisure-time, domestic and commuting), dimensions (e.g., type, rate, intensity and duration) and different modes of assessment, either quantitative or qualitative or a combination of both [10, 16, 18–23]. Use of different physical activity assessment methods in different studies makes it difficult to compare findings across populations [24]. There is also no consensus on a standard threshold level for physical activity level in relation to health benefits. While the dose-response relationship between physical activity and risk of diabetes and other diseases has been demonstrated, there is paucity of literature where different measures of assessing physical activity have been applied to the same population. In this study, the aim was to demonstrate the use of different self-reported measures of the main physical activity domains and their association with the risk of T2D in the Malmö Diet and Cancer Study (MDCS) cohort.

## Methods

### Study design, setting and population

The MDCS is a population-based prospective cohort established in Malmö, Sweden, between 1991 and 1996 when baseline assessment was done. Participants included women born between 1923 and 1950, and men born between 1923 and 1945 who were living in Malmö in southern Sweden whose population was about 230,000 at the time of initiation of the study. The study was approved by the Ethics Committee at Lund University. Baseline information was collected over two visits. In the first visit, participants were taken through the objectives of the study, instructed on how to fill in the detailed self-administered lifestyle questionnaire, dietary questionnaire and 7-day diary (for dietary assessment and medication use) and asked to give informed consent. Blood pressure and anthropometric measures were done and blood samples were drawn by project nurses [25]. Participants were asked to fill in the questionnaires at home. During the second visit, the information in the questionnaires was cross-checked with the participants and missing information were filled in and an interview on dietary habits was conducted. Details about recruitment of participants and details of the visits have been extensively described elsewhere [26–28].

### Eligibility and exclusion criteria

**Fig. 1:**
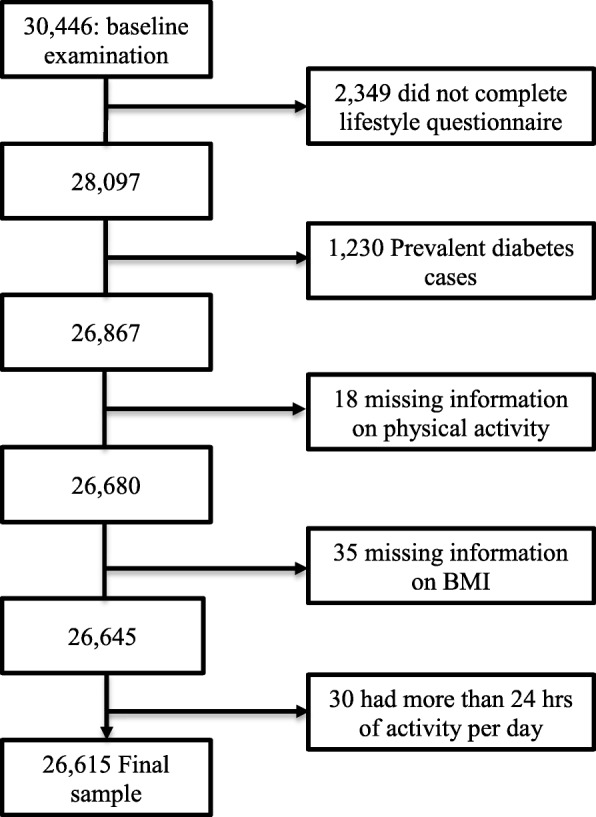
Flow chart of final sample ascertainment

Out of the 74,318 individuals invited, 30,446 participants took part in the baseline examination, and these individuals have been shown to be approximately representative of the population at the time of recruitment [27]. We excluded subjects who did not complete the lifestyle questionnaire (*n* = 2349), those who had prevalent diabetes (both T2D and type 1 diabetes) or used diabetes medication at enrollment (self-reported or identified by registry linkages) (*n* = 1230), those with missing information on leisure-time physical activity (*n* = 187) and BMI (*n* = 35), and those who reported being active for more than 24 h per day (*n* = 30). The final sample size was 26,615 participants (Fig. 1).

### Outcome assessment

Information on T2D was ascertained from multiple registers: the Swedish National Diabetes Register, the Regional Diabetes 2000 Register, the HBA1c Register, the National Patient Register, the Cause of Death Register, and the Swedish Prescribed Drug Register [25, 26, 29, 30]. In the National Diabetes Register and the Regional 2000 Diabetes Register, a proven diagnosis by a physician based on two measurements of fasting plasma glucose of ≥7.0 mmol/L was required [31]. The tenth edition of International Classification of Diseases (ICD-10) codes E10-E14 and O244-O249 were used to identify cases in the National Patient Register and the Swedish Cause of Death register while Anatomic Therapeutic Chemical classification (ATC) code 10 was used to identify diabetes patients in the prescribed drug register [32]. Further notification of cases was obtained from re-examination of subgroups of the cohort participants and during screenings for the Malmö Preventive Project in which participants overlapped between the studies [33]. Incident cases were defined as individuals with a fasting blood glucose of ≥6.5 mmol/L or fasting plasma glucose ≥7.0 mmol/L (verified with plasma or OGTT in subsequent examination), ≥11 mmol/L two hours after OGTT, those who were taking diabetes medication (A10 drugs) or who reported having diabetes in the questionnaire. End of follow-up was December 31, 2014.

### Assessment of physical activity

We used four different domains of physical activity: leisure-time physical activity, occupational physical activity, domestic physical activity and total physical activity level (PAL total). Participants were asked in the questionnaire to indicate minutes spent per week on each of seventeen activities, for instance walking, cycling, and playing soccer (Supplemental Table 1), during each of the four seasons in the preceding year. Leisure-time physical activity in MET-hours per week was computed by multiplying time (hours) spent on each activity by the respective metabolic equivalent task (intensity) factor (MET) (Supplemental Table 1). One MET is described as the metabolic intensity when a person is at rest [34]. Occupational physical activity for those who were currently employed was estimated by multiplying the amount of time spent at work with the intensity levels described by Mattisson et al. [25]; four or five different predefined levels were available depending on the version of the questionnaire. Domestic physical activity was estimated through the questionnaire where participants were also asked to report on the hours per week spent on household chores including shopping. The duration was multiplied by a factor of 2.5 METs to constitute domestic physical activity. For total physical activity we also took time for sleeping, self-care and passive time into account. Sleeping time was taken as 7.3 h per day based on the median reported in a MDCS sub-sample of both men and women in all age groups and was thus adopted for all participants in this study [25]. The intensity factor used for sleeping was 0.9 METs. Self-care estimated to one hour per day for all participants with an intensity factor of 1.6 METs. Amount of passive time (hours) was calculated by subtracting from 24 the total time (hours) spent on all recorded activities (active time) per day. An intensity factor of 1.3 METs was used for passive time. To compute the hourly physical activity level (PAL), total physical activity in MET-hours per day (sum of all activities per day) was divided by 24. To compute physical activity in minutes per week, time reported in hours per week for each of the domains was multiplied by 60.

### Covariate assessment

Information on lifestyle factors was collected from the baseline questionnaire. Smoking status was categorized into smokers, ex-smokers and never smokers. Education status was categorized in five categories (elementary; primary and secondary; upper secondary; further education without a degree and university degree). Body weight (kilograms) and height (centimeters) were measured by trained nurses. Body mass index (BMI) was calculated as kilograms per square of height in meters (kg/m^2^) and individuals were categorized as normal weight (BMI < 25 kg/m^2^), overweight (BMI 25–30 kg/m^2^) and obese (BMI ≥ 30 kg/m^2^). Dietary intake was assessed with a validated modified diet history method combining a 168-item diet history questionnaire covering the past year and a seven-day (consecutive) food diary that included records of cooked meals, nutrient supplements and cold beverages [35, 36]. The total energy intake in kcal/day was estimated by combining the average daily food intake with data from the nutrient database (PC KOST-93 of the Swedish National Food Agency). Participants were categorized into three groups according to a diet risk score that was created by classifying individuals according to intake levels of unfavorable foods (processed meat and sugar sweetened beverages), and favorable foods (whole grains and coffee) [37]. Alcohol intake was reported in the 7-day food diary. The group that reported no alcohol consumption at all during the 7 days and indicated no consumption during the last year in the questionnaire were classified as zero-consumers in both men and women. The other participants were grouped into 4 groups using sex-based cut-points (men: < 20, 20–40 and > 40 g/d. Women: < 15, 15–30 and > 30 g/d). Potential mis-reporters of energy intake (and thereby also potential mis-reporters of physical activity, either under-reporters or over-reporters) were identified by comparing the estimated PAL values with reported energy intake divided by the basal metabolic rate (BMR). Participants with an energy intake to BMR ratio below the lower 95% confidence limit were classified as low energy reporters (i.e. high physical activity reporters), those within the confidence limits were classified as adequate reporters and those above the upper 95% confidence limit were classified as high energy reporters (i.e. low physical activity reporters) [25].

### Statistical analysis

Analysis was carried out using Stata statistics software version 13 (StataCorp LP, College Station, TX). *P* < 0.05 was used as the significance level and all tests were two sided. Each measure of physical activity was categorized into equal quintiles (five groups) except for occupational physical activity where quartiles (four categories for those that were employed) were used due to the large number of individuals (*n* = 10,813) being retired or reported as being unemployed. Participant characteristics across the physical activity groups were investigated. Cox proportional hazards regression was used to model the association between each of the four measures of physical activity and the risk of incident T2D using the duration of follow-up as the underlying time-scale. Individuals were censored at date of emigration or loss to follow-up, death or date until last day of follow-up, whichever occurred first. We created four multivariable models based on sequential adjustment of covariates. The basic model was adjusted for age and sex. The second model was additionally adjusted for smoking, education level and alcohol intake. The third model was additionally adjusted for total energy intake and diet risk score and the final model was additionally adjusted for BMI. The proportional hazards assumption was assessed using the Schoenfeld test. Two variables, age and BMI, violated the proportional hazards assumption. Stratification by these variables did not influence results. Several sensitivity analyses were carried out: 1) excluding potential mis-reporters of physical activity (over-reporters and under-reporters of energy intake), 2) using at least two sources of recorded diabetes diagnosis, 3) excluding participants with a follow-up time of two years or less, and 4) substituting different measures of adiposity (waist circumference, waist-hip ratio and body fat percentage) for BMI. We also examined heterogeneity of effect by testing the multiplicative interaction between physical activity measures and BMI, diet risk score or gender on risk of T2D.

To assess the dose-response relationship between physical activity and risk of T2D, we used restricted cubic splines to model each of the measures (except occupational) of physical activity. Continuous variables used to model the best fitting splines were rounded off to one decimal place and five knots placed at 0, 20th, 40th, 60th and 80th percentiles used in each case. For occupational physical activity, three knots (0, 33rd, and 66th percentiles) were used because of the high number of participants who reported zero occupational activity. To test for nonlinearity, we used the likelihood ratio test where the model without the splines was nested in the model that included the splines.

## Results

Among the 26,615 participants, walking was the most common leisure-time physical activity (85%), followed by cycling (61%) and taking stairs (53%). Orienteering, soccer and table tennis were the least common (0.4, 1 and 1.1% respectively) (Supplemental Table 1). Women had a higher total activity level (PAL) than men; 1.62, (SD = 0.28) and 1.59 (SD = 0.35) for men and women, respectively. However, men had on average more leisure-time MET-hours/week (30.5, SD = 21.9 and 32.3, SD = 26.0) for men and women, respectively) and more minutes-per-week of leisure-time physical activity than women (467 min/week, SD = 431 and 482 min/week, SD = 373 for men and women, respectively). The two measures of leisure-time physical activity (MET-hrs and minutes per week) were highly positively correlated (r = 0.98). Total PAL was highly correlated with occupational physical activity (r = 0.85); however, occupational physical activity was weakly negatively correlated with leisure-time physical activity (r = − 0.06) and domestic physical activity (r = − 0.19) (Supplemental Table 2).

Table 1 shows participants’ characteristics according to groups of leisure-time physical activity in MET-hours per week, PAL, occupational physical activity and domestic physical activity. Both high leisure-time physical activity and total physical activity were correlated with lower BMI and body fat percentage. However, while high leisure-time physical activity was associated with higher age and included a higher number of participants that were retired, total physical activity was associated with lower age and the highest quintile included no retired participants. Overall, persons in higher quantiles of physical activity reported higher energy intake per day and tended to over-report physical activity levels compared to those in lower quantiles (Table 1).

**Table 1:** Participant characteristics according to groups of physical activity (MET-hrs per week) in the MDCS cohort

Characteristics	Leisure-time physical activity			Total physical activity (PAL)			Ocupational physical activity				Domestic Physical Activity		
	Quintile 1 (< 13)	Quintile 3 (22–31)	Quintile 5 (> 46)	Quintile 1 (< 1.39)	Quintile 3 (1.51–1.89)	Quintile 5 (> 2.53)	Quartile 1 (0)	Quartile 2 (1.5–60)	Quartile 3 (60–120)	Quartile 4 (> 120)	Quintile 1 (< 12.5)	Quintile 3 (25–37.5)	Quintile 5 (> 52.5)
Age, y	57.7 (7.4)	57.6 (7.6)	58.9 (7.9)	61.5 (7.7)	58.9 (8.1)	54.2 (4.6)	63.6 (6.8)	56.5 (7.0)	57.6 (6.2)	54.0 (5.4)	58.3(7.1)	57.2(7.4)	59.7 (8.1)
BMI, kg/m^2^	26.2 (4.3)	25.5 (3.8)	25.4(3.7)	26.3 (4.1)	25.4 (3.9)	25.5 (3.7)	26.1(4.04)	25.8(4.08)	25.2(4.09)	25.3(3.77)	26.1 (3.6)	25.5 (3.9)	25.6 (4.2)
Body fat %	27.7 (7.2)	26.9 (6.8)	25.8 (6.9)	26.3 (7.4)	27.5 (6.7)	25.3 (6.8)	27.5(7.25)	27.5(6.69)	28.6(6.23)	26.2)6.68)	22.3 (6.2)	26.8 (6.8)	30.1 (5.7)
Energy intake, kcal/d	2223 (665)	2258 (622)	2377 (696)	2230 (641)	2250 (637)	2434 (689)	2226(644)	2186(730)	2213(603)	2320(656)	2534 (691)	2266 (645)	2117 (558)
Women	60%	63%	57%	47%	73%	49%	60.22%	70.42%	81.36%	61.48%	16.32%	63.67%	91.87%
Smokers	34%	28%	26%	30%	25%	29%	26.32%	22.54%	22.71%	29.93%	27.91%	29.79%	25.29%
Alcohol abstainers	9%	5%	6%	9%	7%	3%	9.3%	7.75%	8.14%	3.75%	4.64%	5.01%	9.05%
High alcohol consumers	5%	4%	5%	5%	3%	6%	3.5%	1.4%	4.41%	4.85%	6.46%	4.81%	2.39%
Lowest education	49%	38%	41%	50%	41%	42%	53.2%	41.9%	39.7%	33.3%	44.66%	37.22%	45.71%
University degree	11%	16%	15%	10%	16%	14%	8.1%	16.2%	16.6%	18.9%	13.33%	16.68%	11.14%
Employed	62%	64%	52%	22%	49%	100%	1.03%	0.89%	1.86%	96.22%	69.17%	64.51%	40.18%
Retired	31%	29%	40%	64%	2%	0%	31%	29%	40%	0%	4.52%	6.67%	12.17%
High diet risk	20%	16%	16%	19%	16%	15%	18.76%	19.01%	14.58%	15.20%	19.25%	15.45%	16.45%
Over-reporters of physical activity	15%	14%	19%	5%	16%	22%	7.50%	9.15%	8.47%	20.75%	12.6%	14.89%	17.2%

### Leisure-time physical activity and risk of T2D

**Fig. 2:**
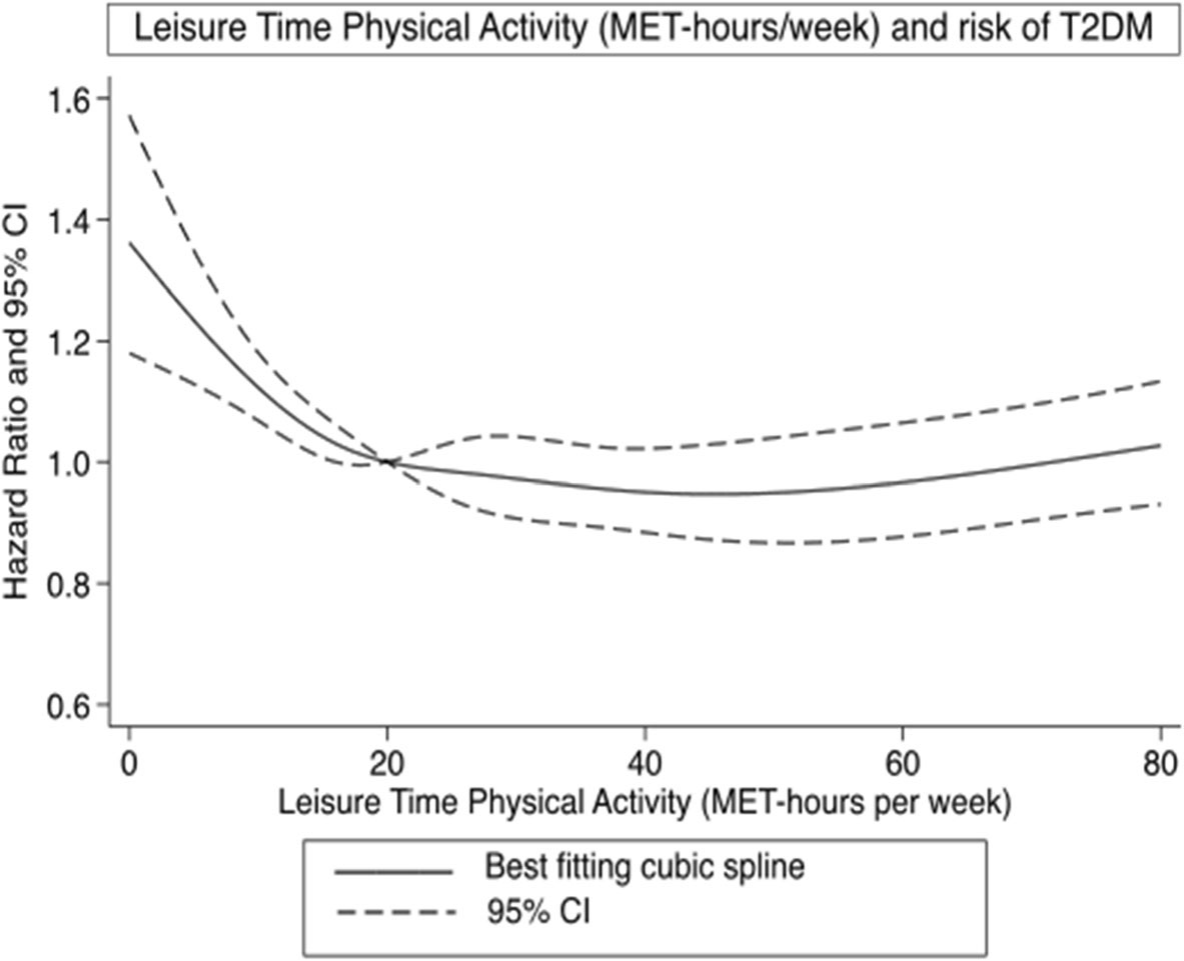
Restricted cubic splines dose-response relationship between leisure-time physical activity and risk of type 2 diabetes. Dashed lines represent 95% CI and solid line represents hazard ratios for estimates obtained from regression of restricted cubic splines (5 knots.) Leisure-time physical activity was truncated at 80 MET-hours per week and reference set at 20 MET-hours per week. P_non-linearity_ < 0.001

In total, 3791 T2D cases were identified during 461,440 person-years of follow-up. Leisure-time physical activity (MET-h/week) was significantly inversely associated with risk of T2D. Introducing BMI into the model attenuated the association; however, the association remained statistically significant. Dose-response relationship modelling using restricted cubic splines showed a curvilinear relationship between leisure-time physical activity and T2D risk. The risk of T2D declined with increase in leisure-time physical activity up to approximately 22 MET-hrs/week beyond which it levelled out (Fig. 2). There was a 22% risk reduction in the fourth quintile (31–46 MET-hours/week) compared with the first quintile. The risk reduction was slightly weaker (13% risk reduction), but still statistically significant, in the group with the highest leisure-time physical activity level (Table 2). Similar results were observed for leisure-time physical activity in minutes per week. We observed 28% (95% CI: 16–43%) increased risk of developing T2D in the group that reported less than 100 min/week of leisure-time physical activity compared with the group that reported more than 300 min/week (Supplemental Table 3).

**Table 2:** Association between measures of physical activity and risk of type 2 diabetes in the MDCS cohort

Physical activity measure	HR (95% CI)				
**Leisure-time physical activity (MET-hrs/wk)**	**< 13**	**13–22**	**22–31**	**31–46**	**> 46**
Cases/Person-years	959/89311	757/92258	704/93747	658/94441	713/91683
Basic model	1.0	0.76(0.69–0.84)	0.70(0.64–0.77)	0.64(0.58–0.71)	0.69(0.63–0.77)
Multivariable model	1.0	0.81(0.73–0.89)	0.76(0.69–0.83)	0.69(0.63–0.77)	0.75(0.68–0.83)
Multivariable model incl. BMI	1.0	0.88(0.80–0.97)	0.84(0.76–0.93)	0.78(0.71–0.87)	0.87(0.78–0.95)
**Domestic physical acitivity (METhrs/wk)**	**< 12.5**	**12.5–25.0**	**25.0–37.5**	**37.5–52.5**	**> 52.5**
Cases/Person-years	996/100437	908/111916	605/81706	524/74353	637/80993
Basic model	1.0	0.96(0.88–1.06)	0.99(0.89–1.10)	1.00(0.89–1.13)	1.13(1.00–1.27)
Multivariable model	1.0	0.97(0.89–1.07)	1.00(0.90–1.12)	1.00(0.89–1.13)	1.10(0.98–1.24)
Multivariable model incl. BMI	1.0	1.01 (0.93–1.11)	1.02(0.91–1.14)	1.03(0.91–1.16)	1.11(0.99–1.25)
**Occupational physical activity (METhrs/wk)**	**0**	**1.5–60**	**60–120**	**> 120**	
Cases/Person-years	1587/170537	834/122192	578/73107	774/94037	
Basic model	1.0	0.78(0.71–0.86)	0.89(0.80–0.99)	0.89(0.80–0.98)	
Multivariable model	1.0	0.88(0.80–0.97)	0.99(0.89–1.10)	0.90(0.81–1.00)	
Multivariable model incl. BMI	1.0	0.95(0.86–1.04)	1.03(0.92–1.15)	0.91(0.82–1.00)	
**PAL**_**total**_	**< 1.37**	**1.37–1.45**	**1.45–1.56**	**1.56–1.89**	**> 1.89**
Cases/Person-years	879/85467	724/91261	674/92531	720/95034	794/97147
Basic model	1.0	0.84(0.76–0.93)	0.81(0.73–0.90)	0.86(0.78–0.95)	0.91(0.82–1.01)
Multivariate Model	1.0	0.89(0.80–0.98)	0.86(0.78–0.96)	0.89(0.81–0.99)	0.89(0.80–0.98)
Multivariate model incl. BMI	1.0	0.94(0.85–1.04)	0.92(0.83–1.02)	0.98(0.89–1.09)	0.93(0.84–1.02)

Basic model: adjusted for age and sex. Multivariate model: adjusted for age, sex, smoking, education, alcohol consumption, diet risk score, and total energy intake

### Occupational physical activity and risk of T2D

**Fig. 3:**
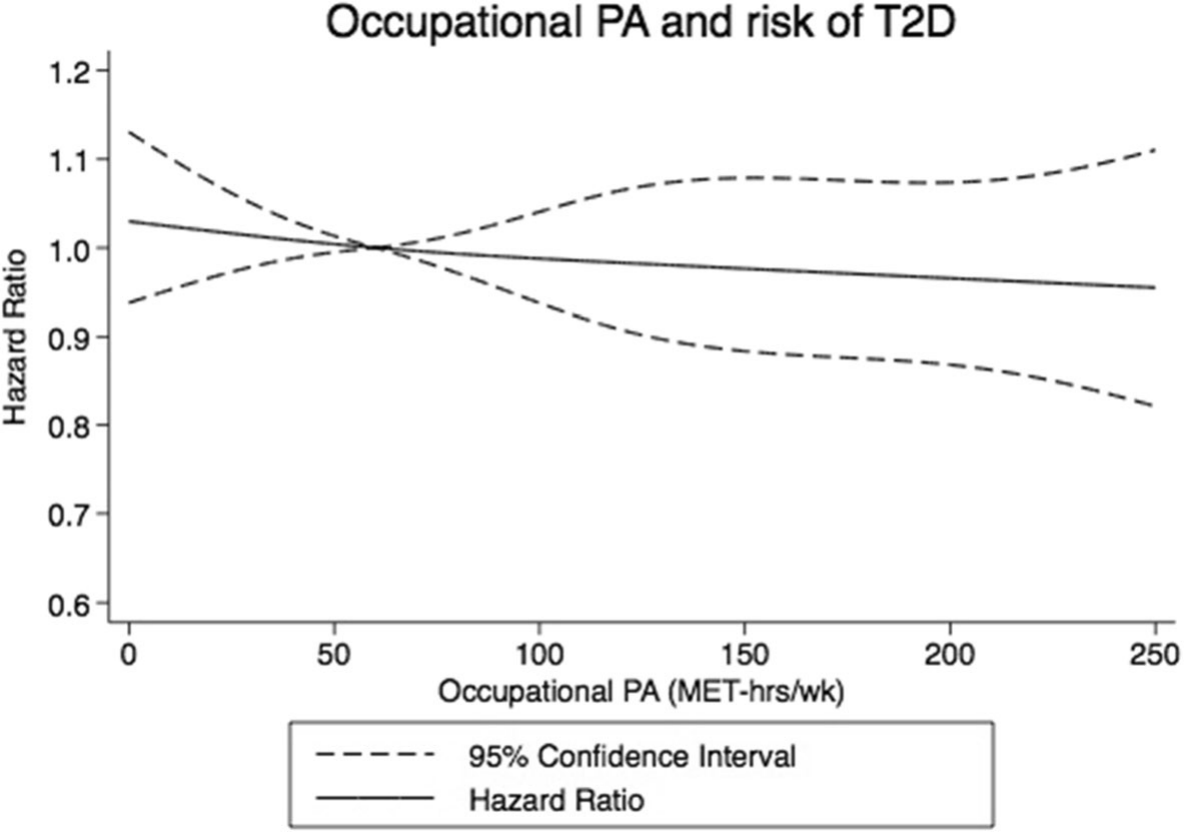
Restricted cubic splines dose-response relationship between occupational physical activity and risk of type 2 diabetes. Dashed lines represent 95% CI and solid line represents hazard ratios for estimates obtained from regression of restricted cubic splines (5 knots.) Occupational physical activity was truncated at 250 MET-hrs/wk. and reference set at 60 MET-hours per week. P_non-linearity_ = 0.84

In the dose-response analysis, we observed a weak inverse association between occupational physical activity and risk of T2D (Fig. 3). The highest vs. the lowest quartile had an HR of 0.91 (95% CI: 0.82–1.00) in the full model (Table 2).

### Domestic physical activity and risk of T2D

**Fig. 4:**
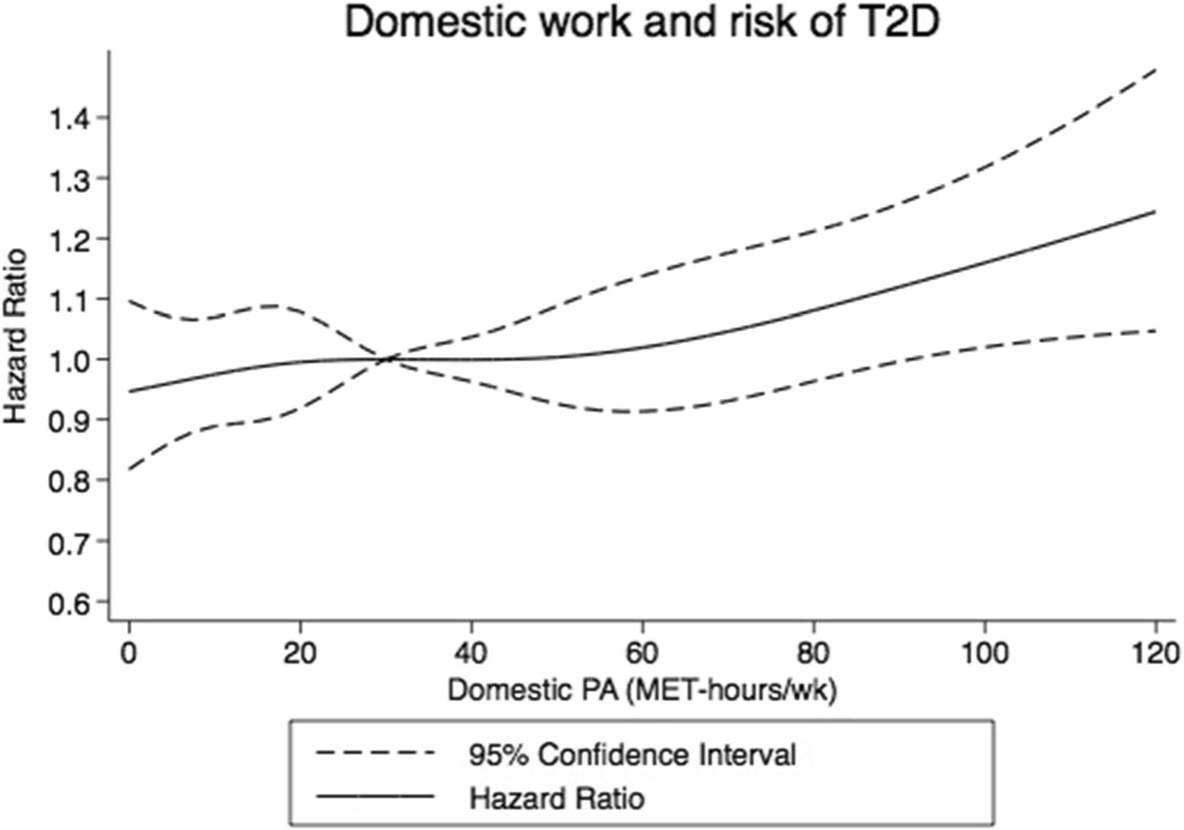
Restricted cubic splines dose-response relationship between domestic physical activity and risk of type 2 diabetes. Dashed lines represent 95% CI and solid line represents hazard ratios for estimates obtained from regression of restricted cubic splines (5 knots.) Domestic physical activity was truncated at 120 MET-hrs/wk. and reference set at 30 MET-hours per week. P_non-linearity_ = 0.72

For domestic physical activity, a modest increased risk of T2D with increase in domestic physical activity was observed (Fig. 4). The highest vs lowest fifth had an HR of 1.11 (95% CI: 0.99–1.25) in the full model (Table 2).

### Total physical activity level (PAL) and risk of T2D

**Fig. 5:**
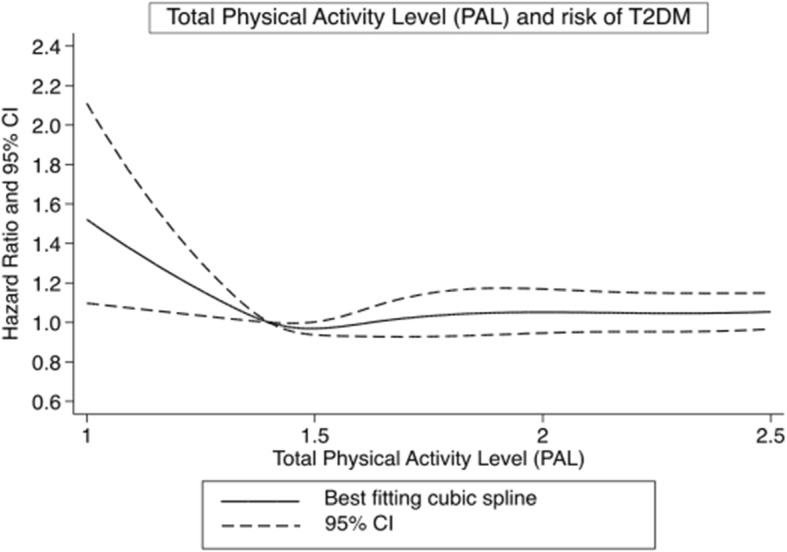
Restricted cubic splines dose-response relationship between total physical activity level (PAL) and risk of type 2 diabetes. Dashed lines represent 95% CI and solid line represents hazard ratios for estimates obtained from regression of restricted cubic splines (5 knots.) PAL was truncated at 2.5 PAL and the reference set at 1.4 PAL. P_non-linearity_ = 0.10

We observed a curvilinear relationship between total physical activity level and risk of incident T2D (Fig. 5). This association was similar, although weaker, as the association observed with leisure-time physical activity. A decrease in risk was observed to about a physical activity level of 1.4 beyond which there was no added advantage. When examining equal quintiles, no significant associations was observed in the final model adjusting for BMI, with a HR of 0.93 (95% CI: 0.84–1.02) in the highest vs lowest group (Table 2).

### Sensitivity and interaction analyses

In sensitivity analyses, i.e. when excluding mis-reporters of physical activity, using cases confirmed from more than one record, substituting other measures of adiposity for BMI or excluding individuals developing T2D within two years after baseline examinations, the results were very similar (Supplemental Tables 4, 5, 6 7). There were no significant interactions between any of the physical activity measures and BMI (all *P* > 0.80), diet risk score (all *P* > 0.77) or sex (all *P* > 0.10) on T2D risk.

## Discussion

In this study, we aimed to assess the association between different domains of self-reported physical activity and risk of T2D in the same population. For leisure-time physical activity, risk reduction was highest comparing those that were inactive with those that were moderately active. Beyond a threshold level of approximately 22 MET-hrs/week or 300 min/week, no additional risk reduction was observed with increase in leisure-time physical activity. Because of the weaker association for occupational physical activity and tendency of increased risk with high domestic physical activity, the association observed between total physical activity (PAL) and risk of T2D was attenuated. We observed that the association between physical activity and risk of T2D was curvilinear for all domains apart from occupational physical activity which was linear. Domestic physical activity was associated with increased risk of T2D while the other domains were associated with decrease in risk. The association between all measures of physical activity used did not differ by gender, BMI level or diet risk score profile.

The proposed benefits of physical activity have been well documented, including reduced levels of inflammation, improved insulin sensitivity (including in diabetics), lipid metabolism and protection against the destruction of pancreatic ß-cells [38]. Physical activity also leads to improved body composition (especially reduction in adiposity) improved cardiorespiratory fitness and reduced risk of cardiovascular diseases, osteoporosis, some cancers and overall mortality [39–41].

Association between physical activity and risk of T2D has been demonstrated in other studies. In a meta-analysis of 55 studies with the aim of quantifying the dose-response association between total physical activity across domains and the risk of diabetes, 28% risk reduction was observed in highly active (> 8000 MET minutes/week, i.e., > 133 MET hours/week) compared to insufficiently active individuals (< 10 MET hours/week). Major health benefits were observed comparing those at lower levels of physical activity to those at moderate levels though there was diminishing returns at levels higher than 50–67 MET hours/week [21]. Other studies have demonstrated the inverse (and curvilinear) association between different forms of leisure-time physical activity and the risk of diabetes, and also reinforced the observation that those who are initially inactive or minimally active have the highest reduction in risk [12, 16, 42].

We found a weak inverse association between occupational physical activity and risk of T2D, with 9% (0–18%) decreased risk in the highest versus lowest quartile. In a meta-analysis of three cohort studies focusing on occupational physical activity, high versus low occupational activity was associated with 15% (95% CI: 8–21%) decreased risk of T2D [16]. However, in the Japanese workers study, no statistically significant association was observed [10]. This could be attributed to the fact that this domain of physical activity may not be adequate confer health benefits especially in persons for whom it is the main form of physical activity who might also, in addition, have other stronger underlying risk factors [43, 44]. In our study, occupational physical activity was negatively correlated with leisure-time and domestic physical activities which may indicate more time spent at work and little time for other physical activity domains. This may partly explain the observed weak association in this population. Further, participants in this study did not report the intensity of occupational physical activity, just the type and duration and therefore this domain warrants further investigation.

The association between domestic physical activity and risk of diabetes observed in this study could be due to inadequate impact of this domain on health benefits or that there may be other underlying risk factors among participants for whom this is the predominant domain of physical activity in this population. Total physical activity level was not significantly associated with T2D in our study. The effects of occupational physical activity may also have contributed to non-significant association with total physical activity level because of their high correlation.

By using cubic splines, we were able to circumvent the shortcomings of categorizing continuous variables and assumptions of linearity and demonstrate that in this population the relationship between physical activity and the risk of T2D was not linear. Our study showed that the lowest risk (not to imply a lack of risk reduction below 300 min per week) was observed among those undertaking between 300 and 600 min of moderate leisure-time physical activity per week. These levels overlap with the WHO recommendation of 150–300 min per week of moderate-intensity aerobic physical activity and/or more than 300 min per week for added health benefits [17]. Our results also indicate that the biggest benefit is accrued by those who initiate physical activity from a prior state of inactivity or low activity, thus the WHO recommendations are valid for this group. A unique feature of our findings is the impact of different physical activity domains on health, in the same population. We demonstrated a different relationship between domestic physical activity and the risk of T2D, compared to the other physical activity domains. The implication is that these inter-domain differences may need to be considered when developing physical activity recommendations. As we mention above, there is need for more studies investigating effects of different physical activity domains in same populations.

The main weakness of the study is the observational design by which we cannot rule out reverse causality, recall bias, and remaining unknown or residual confounding. We were also limited by lack of data on sedentary time. However, a major strength was the use of a population-based cohort with detailed data on the most important potential confounders of this study, and the use of multiple registers to confirm the diabetes outcome. We also used restricted cubic splines to better understand the relationship between T2D and physical activity which was not linear, and included 17 different leisure-time physical activities which were all factored in computing the exposure. The use of METs assumes the same average intensity for each person for a particular activity which is unlikely, but it is nonetheless a robust metric especially for self-reported physical activity data.

## Conclusion

In this study investigating different measures of physical activity in the same population, leisure-time physical activity had strong inverse association with risk of T2D while other measures had weaker associations. Domestic physical activity was associated with increased risk of T2D though this association was weak. In terms of health promotion, the key message from these findings is that persons currently inactive or with very low levels of physical activity may gain more health benefits if they became active or increased their levels of activity respectively.

## Supplementary information

**Additional file 1: Supplemental Table 1.** List of leisure-time physical activities in the MDC cohort. **Supplemental Table 2.** Correlations between different measures of physical activity in the MDCS. **Supplementary Table 3.** HR and 95% CI for association between physical activity and T2D in the MDCS using spline-based cut-points, 1991-1996. **Supplemental Table 4.** Association between measures of physical activity risk of T2D among adequate reporters of energy intake in the MDCS, 1991-1996. **Supplemental Table 5.** Measures of physical activity and risk (HR, 95% CI) of incident T2D in the MDCS adjusted for different measures of adiposity, 1991-1996. **Supplemental Table 6.** Measures of physical activity and risk (HR, 95% CI) of incident T2D excluding the first 2 years of follow-up in the MDCS, 1991-1996. **Supplemental Table 7.** Measures of physical activity and risk (HR, 95% CI) of incident T2D, confirmed from at least two sources in the MDCS, 1991-1996. This file contains additional data relevant to interpretation of the study findings. It includes information on the different types of actual physical activities reported by participants, correlations between the different measures of physical activity and results of the various sensitivity analyses conducted

## Data Availability

All datasets used and/or analyzed during the current study are available from the corresponding author on reasonable request subject to Swedish laws and regulations. The questionnaire can be found on the following website: https://www.malmo-kohorter.lu.se/mkc/datainsamling-mkc.

## Abbreviations

ATC: Anatomic Therapeutic Classification
BMI: Body Mass Index
ICD: International Classification of Diseases
MDCS: Malmö Diet and Cancer Study
MET: Metabolic Equivalent
OGTT: Oral Glucose Tolerance Test
T2D: Type 2 diabetes
WHO: World Health Organization
