# Supplementary material for: Different domains of self-reported physical activity and risk of type 2 diabetes in a population-based Swedish cohort: the Malmö diet and Cancer study
Source: BMC Public Health. 2020 Feb 21;20:261. doi: 10.1186/s12889-020-8344-2 (PMC7035654; doi:10.1186/s12889-020-8344-2)
Supplement: Supplementary file 1 — Additional file 1: Supplemental Table 1. List of leisure-time physical activities in the MDC cohort. Supplemental Table 2. Correlations between different measures of physical activity in the MDCS. Supplementary Table 3. HR and 95% CI for association between physical activity and T2D in the MDCS using spline-based cut-points, 1991-1996. Supplemental Table 4. Association between measures of physical activity risk of T2D among adequate reporters of energy intake in the MDCS, 1991-1996. Supplemental Table 5. Measures of physical activity and risk (HR, 95% CI) of incident T2D in the MDCS adjusted for different measures of adiposity, 1991-1996. Supplemental Table 6. Measures of physical activity and risk (HR, 95% CI) of incident T2D excluding the first 2 years of follow-up in the MDCS, 1991-1996. Supplemental Table 7. Measures of physical activity and risk (HR, 95% CI) of incident T2D, confirmed from at least two sources in the MDCS, 1991-1996. This file contains additional data relevant to interpretation of the study findings. It includes information on the different types of actual physical activities reported by participants, correlations between the different measures of physical activity and results of the various sensitivity analyses conducted [file 12889_2020_8344_MOESM1_ESM.pdf]

SUPPLEMENTAL MATERIAL

Different domains of self-reported physical activity and risk of type 2 diabetes in a population-based Swedish cohort: The Malmö Diet and Cancer Study

Pascal M. Mutie[1], Isabel Drake[1], Ulrika Ericson[1], Stanley Teleka[2], Christina-Alexandra Schulz[3], Tanja Stocks[2], Emily Sonestedt[1]

[1]Department of Clinical Sciences Malmö, Lund University, Malmö, Sweden

[2, 3]Department of Clinical Sciences Lund, Lund University, Lund, Sweden

Corresponding author: Emily Sonestedt, emily.sonestedt@med.lu.se

S1.

Supplemental Table 1. List of leisure-time physical activities in the MDC cohort

				Diabetes status	
				No (n=22824)	Cases (n=3791)
Activity	METs	Number involved in the activity	Minutes/week among those involved (median, IQR)	Involved (%)	Involved (%)
**Moderate intensity activites**					
Walking	3.5	22708	150 (187.5)	85.8%	82.7%
Table tennis	4.0	291	37.5 (75)	1.1%	1.3%
Cycling	4.0	16323	110 (150)	62.2%	56.0%
Gardening	4.0	12395	82.5 (112.5)	46.9%	44.3%
Gymnastics	4.0	6113	45 (56.25)	24.0%	16.6%
Golf	4.5	1986	240 (242.5)	7.7%	6.1%
Badminton	4.5	619	45 (41.25)	2.4%	2.2%
Digging	5.0	5806	30 (45)	21.7%	22.4%
Folk dancing	5.5	1037	90 (90)	4.0%	3.5%
Ballroom dancing	5.5	2705	60 (90)	10.2%	9.8%
Grass cutting	6.0	7973	22.5 (30)	29.9%	30.4%
**Vigorous intensity activities**					
Jogging	7.0	2668	45 (60)	10.6%	6.6%
Swimming	7.0	5251	30 (30)	20.0%	18.1%
Lawn Tennis	7.0	683	60 (75)	2.7%	2.0%
Soccer	8.0	284	45 (71.25)	1.0%	1.5%
Orienteering	9.0	112	30 (48.75)	0.4%	0.4%
Walking stairs	9.0	14142	25 (31.25)	53.8%	49.1%

S2.

Supplemental table 2. Correlations between different measures of physical activity in the MDCS

	LTPA (METhrs/wk)	LTPA (min/wk)	PAL_{total}	Occupational work	Domestic work
LTPA(METhrs/wk)	1.00				
LTPA(min/wk)	0.98	1.00			
PALtotal	0.30	0.27	1.00		
Occupational	-0.06	-0.09	0.85	1.00	
Domestic work	0.06	0.08	0.14	-0.19	1.00

LTPA = Leisure time physical activity
PAL = Physical activity level

S3.

Supplementary Table 3. HR and 95% CI for association between physical activity and T2D in the MDCS using spline-based cut-points, 1991-1996

**Physical activity measure**	**Physical activity levels and Hazard Ratios (95% CI) ‡**			
LTPA min/week	**< 100 min/week**	**100-200 min/week**	**200-300 min/week**	**> 300 min/week**
	1.28 (1.16-1.43)	1.14 (1.04-1.26)	1.07 (0.97-1.17)	1.00
LTPA MET-hrs/wk	**< 7.5 MET-hrs/wk**	**7.5-15 MET-hrs/wk**	**15-22 MET-hrs/wk**	**>22 MET-hrs/wk**
	1.29 (1.17-1.42)	1.13 (1.04-1.24)	1.03 (0.94-1.13)	1.0
PAL_{total}	**< 1.3**	**1.3-1.6**	**1.6-2.0**	**>2.0**
	1.07 (0.93-1.22)	0.96 (0.87-1.02)	0.99 (0.89-1.09)	1.00

‡ Models adjusted for age, sex, smoking, education, alcohol consumption, diet risk score, total energy intake, and BMI.

S4. Sensitivity analyses

4.1 Excluding mis-reporters of energy intake

Supplemental Table 4. Association between measures of physical activity risk of T2D among adequate reporters of energy intake in the MDCS, 1996

**LTPA MET-Hrs/wk**	**<13**	**13-22**	**22-31**	**31-46**	**>46**
Cases/Person-years	743/72270	610/76350	548/77966	519/78491	531/72069
Multiv. Model	1.0	0.89(0.80-0.99)	0.82(0.74-0.92)	0.76(0.68-0.86)	0.86(0.76-0.96)
**Domestic PA METhrs/wk**	**<12.5**	**12.5-25.0**	**25.0-37.5**	**37.5-52.5**	**>52.5**
Cases/Person-years	795/83242	725/92702	460/66989	395/60398	484/64414
Multiv. Model	1.0	1.03(0.93-1.14)	1.01(0.89-1.14)	1.03(0.90-1.18)	1.14(1.00-1.30)
**Occupational PA METhrs/wk**	**0(zero)**	**1.5-60**	**60-120**	**>120**	
Cases/Person-years	1363/149731	739/112085	442/59579	391/54456	
Multiv. Model	1.0	0.94(0.84-1.04)	1.02(0.90-1.15)	0.90(0.78-1.02)	
**PALtotal**	**<1.37**	**1.37-1.45**	**1.45-1.56**	**1.56-1.89**	**>1.89**
Cases/Person-years	879/85467	724/91261	674/92531	720/95034	794/97147
Multiv. Model	1.0	0.94(0.85-1.04)	0.92(0.83-1.02)	0.98(0.89-1.08)	0.93(0.83-1.02)

Multiv.Model: adjusted for age, sex, smoking, education, alcohol consumption, diet risk score, total energy intake, and BMI.

4.2 Replacing BMI with other measures of adiposity

Supplemental Table 5. Measures of physical activity and risk (HR, 95% CI) of incident T2D in the MDCS adjusted for different measures of adiposity, 1996

**LTPA MET-Hrs/wk**	**<13**	**13-22**	**22-31**	**31-46**	**>46**
Cases/Person-years	959/89272	757/92298	704/93709	658/94479	713/91682
Multiv. Model $_{waist}$	1.0	0.90(0.82-0.99)	0.89(0.81-0.99)	0.84(0.76-0.93)	0.93(0.84-1.03)
Multiv. Model $_{WHR}$	1.0	0.86(0.78-0.95)	0.83(0.75-0.92)	0.77(0.70-0.85)	0.80(0.73-0.89)
Multiv. Model. $_{BFP}$	1.0	0.87(0.79-0.96)	0.84(0.76-0.92)	0.79(0.72-0.88)	0.87(0.79-0.96)
**Domestic METhrs/wk**	**<12.5**	**12.5-25.0**	**25.0-37.5**	**37.5-52.5**	**>52.5**
Cases/Person-years	996/100437	908/111916	605/81706	524/74353	637/80993
Multiv. Model $_{waist}$	1.0	1.02(0.93-1.12)	1.02(0.91-1.14)	1.03(0.92-1.17)	1.12(1.00-1.26)
Multiv. Model $_{WHR}$	1.0	0.99(0.91-1.09)	0.99(0.89-1.11)	0.96(0.85-1.08)	1.10(0.98-1.24)
Multiv. Model $_{BFP}$	1.0	0.98(0.89-1.07)	0.99(0.89-1.11)	0.98(0.96-1.22)	1.08(0.96-1.22)
**Occupational PA METhrs/wk**	**0(zero)**	**1.5-60**	**60-120**	**>120**	
Cases/Person-years	1587/170537	834/122192	578/73107	774/94037	
Multiv. Model $_{waist}$	1.0	0.99(0.90-1.09)	1.06(0.95-1.18)	0.95(0.86-1.05)	
Multiv. Model $_{WHR}$	1.0	0.95(0.86-1.05)	1.06(0.95-1.18)	0.94(0.85-1.04)	
Multiv. Model. $_{BFP}$	1.0	0.95(0.86-1.05)	1.06(0.95-1.18)	1.00(0.90-1.10)	
**PALtotal**	**<1.37**	**1.37-1.45**	**1.45-1.56**	**1.56-1.89**	**>1.89**
Cases/Person-years	879/85467	724/91261	674/92531	720/95034	794/97147
Multiv. Model $_{waist}$	1.0	0.99(0.90-1.10)	0.98(0.89-1.09)	1.02(0.92-1.13)	1.00(0.91-1.11)
Multiv. Model $_{WHR}$	1.0	0.96(0.87-1.06)	0.89(0.81-0.99)	0.96(0.86-1.06)	0.93(0.84-1.03)
Multiv. Model $_{BFP}$	1.0	0.96(0.87-1.06)	0.95(0.86-1.06)	1.00(0.91-1.11)	1.02(0.92-1.13)

Waist = Waist circumference in centimeters
WHR – Waist to Hip Ratio
BFP – Body fat percentage
Multiv. Model: adjusted for age, sex, smoking, education, alcohol consumption, diet risk score, total energy intake, and the stated measure of adiposity.

4.3 Excluding the first 2 years of follow-up

Supplemental Table 6. Measures of physical activity and risk (HR, 95% CI) of incident T2D excluding the first 2 years of follow-up in the MDCS, 1996

**LTPA MET-Hrs/wk**	**<13**	**13-22**	**22-31**	**31-46**	**>46**
Cases/Person-years	959/89272	757/92298	704/93709	658/94479	713/91682
Multiv. Model	1.0	0.91(0.82-1.00)	0.86(0.78-0.95)	0.80(0.72-0.88)	0.88(0.80-0.98)
**Domestic METhrs/wk**	**<12.5**	**12.5-25.0**	**25.0-37.5**	**37.5-52.5**	**>52.5**
Cases/Person-years	923/100291	840/111780	553/81603	482/74278	591/80910
Multiv. Model	1.0	1.01(0.91-1.11)	1.00(0.89-1.12)	1.01(0.89-1.14)	1.11(1.46-1.77)
**Occupational PA METhrs/wk**	**0(zero)**	**1.5-60**	**60-120**	**>120**	
Cases/Person-years	1426/170188	787/122107	540/73050	729/93970	
Multiv. Model	1.0	0.96(0.87-1.06)	1.04(0.93-1.16)	0.93(0.83-1.03)	
**PALtotal**	**<1.37**	**1.37-1.45**	**1.45-1.56**	**1.56-1.89**	**>1.89**
Cases/Person-years	804/85256	650/91173	629/92434	672/94946	744/97070
Multiv. Model	1.0	0.91(0.82-1.01)	0.92(0.83-1.03)	0.98(0.88-1.09)	0.92(0.83-1.02)

Multiv.Model: adjusted for age, sex, smoking, education, alcohol consumption, diet risk score, total energy intake, and BMI.

4.4. Using at least 2 sources to confirm diabetes

Supplemental Table 7. Measures of physical activity and risk (HR, 95% CI) of incident T2D, confirmed from at least two sources in the MDCS, 1996

**LTPA MET-Hrs/wk**	**<13**	**13-22**	**22-31**	**31-46**	**>46**
Cases/Person-years	771/89272	594/92298	553/93709	514/94479	543/91683
Multiv. Model	1.0	0.87(0.79-0.97)	0.84(0.75-0.94)	0.78(0.70-0.86)	0.85(0.76-0.95)
**PAL_{total}**	**<1.37**	**1.37-1.45**	**1.45-1.56**	**1.56-1.89**	**>1.89**
Cases/Person-years	719/85411	566/91317	532/92531	565/95034	593/97147
Multiv. Model	1.0	0.91(0.82-1.02)	0.91(0.81-1.02)	0.95(0.85-1.07)	0.83(0.74-0.93)
**Domestic METhrs/wk**	**<12.5**	**12.5-25.0**	**25.0-37.5**	**37.5-52.5**	**>52.5**
Cases/Person-years	789/100437	723/111916	454/81706	408/74353	503/80993
Multiv. Model	1	1.03 (0.93-1.14)	0.97(0.86-1.10)	1.03(0.90-1.18)	1.13(0.99-1.29)
**Occupational PA METhrs/wk**	**0(zero)**	**1.5-60**	**60-120**	**>120**	
Cases/Person-years	1244/170537	667/122192	468/73107	580/94038	
Multiv. Model	1	0.95(0.86-1.06)	1.03(0.92-1.17)	0.83(0.74-0.93)	

Multiv.Model: adjusted for age, sex, smoking, education, alcohol consumption, diet risk score, total energy intake, and BMI.
